# Post-fast refeeding: rise of intestinal stemness and mutagen-induced cancer risk through polyamine metabolism

**DOI:** 10.1038/s41392-024-02038-1

**Published:** 2024-11-11

**Authors:** Sarah Brunner, Klaus-Peter Janssen, Josef Ecker

**Affiliations:** 1 Institute of Clinical Chemistry and Laboratory Medicine, Functional Lipidomics and Metabolism Research, University Hospital Regensburg, Regensburg, Germany; 2 Technical University of Munich, School of Medicine and Health, Dept. of Surgery, 81675 Munich, Germany

**Keywords:** Gastrointestinal cancer, Cell biology

An increased intestinal stem cell activity following post fast-refeeding is beneficial for regeneration, but too much could increase the risk for cancer in mice, as reported in a recent study by Imada and colleagues published in *Nature*.^1^ In consequence, fast-refeeding cycles should be thoroughly planned, when dietary strategies are considered for regeneration to minimize cancer risks.

For many years, fasting interventions like low-calorie diets and intermittent fasting are thought to lengthen lifespan, enhance tissue regeneration and have inhibitory effects on tumor growth. In a previous study, the authors showed that short and acute fasting boosts the regenerative capacity of intestinal stem cells (ISCs).^2^ ISCs are among the most actively dividing cells and they are essential for the constantly renewing intestinal epithelium, which is directly exposed to numerous food components. Upon fasting ISCs shift their major energy source from carbohydrates to lipids by activating fatty acid oxidation pathways. However, many details are still unclear, including the very fundamental question: Does fasting itself or food intake shortly afterwards drive regeneration? To answer this question, various genetic mouse models were fasted for 24 h, fasted for 24 h and refed, or left with ad libitum access to food (control group). Those included a reporter line allowing to trace lineage Lgr5^+^ ISCs that are located at the crypt base, first described almost two decades ago by the group of Hans Clevers.^3^ Lgr5^+^ ISCs are supported by epithelial, stromal and immune cell niches and regulated by the diet and the gut microbiome.

Most importantly, the researchers could show that many of the fasting benefits in the intestine take place at the refeeding cycle. Crypt cell proliferation and function dramatically increased in homeostasis as well as radiation-induced injury due to induction of the nutrient-sensing PI3K and mTORC1 pathways. Ornithine aminotransferase, a mitochondrial enzyme producing the non-proteogenic amino acid ornithine, was among the most strongly upregulated genes in refed ISCs. Ornithine in turn, is used for synthesis of polyamine, which stimulates global protein translation via the eukaryotic translation initiation factor 5 A. High amounts of cellular protein mass are required to build new, more differentiated and specialized intestinal cell types necessary for tissue regeneration. The mechanistic details were deciphered in vivo with the use of rapamycin as mTORC1 inhibitor, genetic mouse models allowing a tamoxifen-inducible intestine-specific activation or ablation of mTORC1 that were fast and refed in combination with single-cell RNA sequencing and mass spectrometry-based analysis of polyamine and ornithine metabolism. Ornithine-triggered de novo synthesis of polyamines was verified applying stable isotope labeling in crypt organoids.

The downside of the extremely high division rates of ISCs, which also makes them special among other adult stem cells, is their susceptibility to become precancerous cells. This holds the possibility that refeeding does not only boost regeneration, but simultaneously initiates tumor formation. Indeed, the team around the senior author Ömer Yilmaz determined an increased risk for intestinal cancer development after refeeding, but only in combination with a defined genetic cancer initiating mutation. Post-fast refeeding of mice having a Cre-mediated loss of the tumor suppressor APC in Lgr5^+^ ISCs, significantly amplified the development of adenomatous polyps and ISC tumorigenesis, which could be dampened by inhibition of mTORC1-mediated polyamine and protein synthesis. It would be valuable to validate these findings with other mouse models of intestinal cancer, inflammation-driven or gnotobiotic, having different magnitudes of tumor development. The model used in this study is based on an extremely strong tumor-induction model, since the functional loss of both “floxed” APC alleles is induced throughout the intestinal tract and in basically every Cre-expressing stem cell. Refeeding gave rise to tumors in the benign adenoma stage, but a progression to the carcinoma stage was not described, most likely due to the limitations of the used mouse model.

**Fig. 1:**
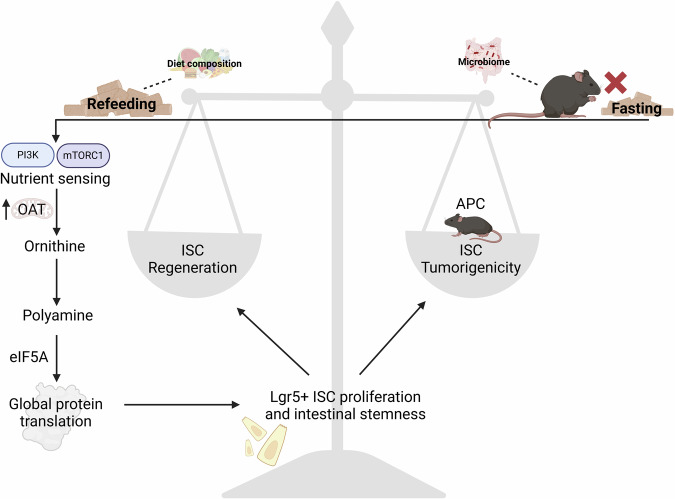
Balance between intestinal stemness and tumorigenesis after post-fast refeeding. In mice, refeeding after fasting increases ISC regeneration by an increased ornithine- and polyamine-dependent protein synthesis initiated via mTORC1 and eIF5A. This response includes the development of tumors in the benign adenoma stage, if the tumor suppressor APC is knocked out in Lgr5^+^ ISCs. Intestinal tumor development and ISC stemness are influenced by the composition of the diet and the gut microbiome, which might be targeted to improve tissue regeneration without increasing cancer risks. Created with BioRender.com

In a nutshell, these findings reveal that fasting and refeeding represent two distinct metabolic states as well as that there is a fine line between fast-refeeding driven stem cell proliferation and tumor formation (Fig. 1). Diet is a provider of cellular energy and structural components are needed to build biomass for proliferating ISCs and cancer cells, supported by enrichment of diet-derived poly-unsaturated lipids in APC^1368N^ mice developing adenomatous and carcinomatous lesions in the intestinal tract as well as in human colorectal cancer tissue.^4^ Obviously, in humans, the scenario is more complicated than in mice, and further studies are needed to determine whether fasting-eating has similar effects. Nevertheless, the findings presented by Imada and coworkers could be relevant for patients, who undergo radiation treatment or have intestinal injuries, where polyamine availability could be increased to augment intestinal repair. Either through direct supplementation, indirectly via nutritional strategies, or even by manipulation of the gut microbiome. Both, diet and the microbiome are known to have significant impacts on stemness and tumorigenicity of intestinal progenitor cells.^5^ In addition, these data suggest to carefully consider the planning of dietary strategies for regeneration to avoid unnecessary cancer risks.
